# T-Cell Subtypes and Immune Signatures in Cutaneous Immune-Related Adverse Events in Melanoma Patients under Immune Checkpoint Inhibitor Therapy

**DOI:** 10.3390/cancers16061226

**Published:** 2024-03-20

**Authors:** Magdalena Absmaier-Kijak, Caterina Iuliano, Susanne Kaesler, Tilo Biedermann, Christian Posch, Knut Brockow

**Affiliations:** 1 Department of Dermatology and Allergy Biederstein, School of Medicine, Technical University of Munich, 80802 Munich, Germany; magdalena.absmaier@tum.de (M.A.-K.); caterina.iuliano@tum.de (C.I.); susanne.kaesler@tum.de (S.K.); sekretariat.derma@mh.tum.de (T.B.); christian.posch@tum.de (C.P.); 2 Department of Dermatology, Clinic Hietzing and Ottakring, 1130 Vienna, Austria; 3 School of Medicine, Sigmund Freud Private University Vienna, 1020 Vienna, Austria

**Keywords:** cancer, melanoma, immunotherapy, immune-related adverse events, immune checkpoint inhibitors, T cells, cutaneous side effects

## Abstract

**Simple Summary:**

Immune-related adverse events (irAEs) as responses to immunotherapy are frequent and a burden to patients. While cutaneous irAEs (cutAEs) are a common side effect, some are linked to better overall survival and clinical responses to immunotherapy in patients. There are emerging reports on cutaneous side effects of immunotherapy; however, in-depth analysis of the underlying immunological signatures is rare. In this pilot study, we analyzed the T-cell signatures of cutAEs in melanoma patients undergoing immunotherapy and detected an evident shift towards a Th17-driven inflammatory profile. We identified distinct cytokine signatures specific for urticaria and T-cell-mediated cutAEs, as well as an increase in IL-10 in non-responders during cutAEs.

**Abstract:**

Immune checkpoint inhibition (ICI) improves outcomes in melanoma patients, but associated T-cell activation frequently leads to immune-related cutaneous adverse events (cutAEs). To dynamically identify T-cell subtypes and immune signatures associated with cutAEs, a pilot study was performed in stage III-IV melanoma patients using blood samples for flow cytometry and cytokine analysis. Blood samples were taken from patients before initiation of ICI (naive), at the onset of a cutAE, and after 6 months of ICI treatment. Overall, 30 patients were treated either with anti-PD1 monotherapy or with anti-PD-1/anti-CTLA-4 combination therapy. Flow cytometry analysis of PBMCs showed that ICI induced an overall shift from a Th2 towards a Th1 profile. Twelve patients (40%) developed cutAEs, which were associated with increased Th22 cells and Th17 cells, supported by a tendency to have elevated Th17/Th22-associated cytokines such as IL-17A, IL-22 and IL-23 levels in the plasma. Cytokine signatures specific for urticaria and T-cell-mediated cutAEs were identified in the plasma of patients by a bead-based assay. IL-10 was elevated in non-responders and, interestingly, during cutAEs. In conclusion, we identified distinct immune signatures based on the Th17/Th22 pathway in cutAEs, both in PBMCs and plasma. In addition, our finding of upregulated IL-10 during cutAEs supports the notion of treating these patients early and adequately to avoid implications for the overall outcome.

## 1. Introduction

Immune checkpoint inhibition (ICI) targeting the programmed cell death protein 1 (PD-1), programmed cell death protein ligand 1 (PD-L1), and cytotoxic T-lymphocyte-associated protein 4 (CTLA-4) has dramatically improved outcomes in melanoma patients [1,2]. These proteins are crucial for maintaining the immunological equilibrium: CTLA-4 regulates the early T-cell-associated immunologic activation, while PD-1 and PD-L1 help to regulate the late T-cell activation in the peripheral tissues [3,4]. By blocking these inhibitory pathways, ICI induces an enhanced T-cell-mediated response to eliminate tumor cells. However, this activation of the immune system leads to the induction of immune-related adverse events (irAEs) that can mimic autoimmune disorders [5]. Even though the physical and psychological burden of irAEs in patients can be high, the development of certain irAEs is linked to a better overall prognosis [6,7]. The organ most often affected is the skin, and cutaneous irAEs (cutAEs) are amongst the earliest irAEs to arise in patients receiving ICI [8,9]. Thus, assessing clinical presentations and understanding the pathology of cutAEs is fundamental for the improvement of management strategies. CutAEs can present in various forms and mimic inflammatory and autoimmune skin disorders [10]. About 30% of patients develop a cutAE to ICI monotherapy and even more to anti-PD-1/anti-CTLA-4 combination therapy [11]. Melanoma patients present with a significantly higher risk of cutAEs compared to patients with other solid malignancies, emphasizing the need to understand cutAEs in melanoma patients [12]. The spectrum of possible cutAEs is very broad. Thus far, cutAEs have been mostly studied using general terms without being systemically delineated in dermatologic terms [13]. In the literature, skin rashes (unspecified or maculopapular), itching and vitiligo are most frequently reported, but eczematous, psoriasis-like and lichenoid skin changes, acneiform exanthemas, alopecia and autoimmune skin diseases such as bullous pemphigoid are also described [14]. Antitumor responses, but also cutAEs, are initiated by T-cell activation; however, the immune phenotype by which ICI might promote cutAEs remains unknown. The objective of this study was to decipher the underlying immunological differences between different manifestations of cutAEs in melanoma patients and to determine whether a common denominator can be determined that will impact clinical treatment. For this, a pilot study was conducted at the department of Dermatology at TUM School of Medicine and Health, and an in-depth analysis of the immune profiles of cutAEs was performed. We focused on the analysis of T-cell subsets and their associated cytokines in patients with and without cutAEs and found that ICI induced an overall shift from a Th2 towards a Th1 profile. Analysis of the immune state during a cutAE showed a distinct immune signature based on the Th17/Th22 pathway, with further identification of IL-10 as a biomarker with potential functional implications, increasing our understanding of the pathophysiology of cutAEs.

## 2. Materials and Methods

### 2.1. Study Design and Patients

A total of 30 patients suffering from stage III/IV malignant melanoma were enrolled in this exploratory prospective non-interventional study. The study protocol was approved by the Ethics committee at the Technical University of Munich (reference number: 2023-611-S-SB, 23 November 2023) and the rules of the Declaration of Helsinki were followed. Informed consent was obtained from all subjects involved in this study.

For patient characterization, the clinical parameters age and gender were noted down in addition to the tumor-specific variables melanoma subtype and site, Breslow thickness in mm, BRAF mutation status, PD-L1 expression and sites involved in metastasis, which were determined by histology of tumor samples (FFPE-fixed skin biopsies). Included patients were naive to ICI therapies, but previous treatments with targeted therapies (BRAF/MEK inhibition), interferon alpha, and radiotherapy followed by disease progression were allowed. In addition, one patient also received 3 chemotherapy cycles with dacarbazine 15 months prior to study inclusion. More detailed inclusion and exclusion criteria are presented in Supplementary Table S1.

According to the clinical setting and after consulting an interdisciplinary tumor board, the patients were treated either with anti-PD-1 monotherapy (in therapeutic settings or after complete resection in adjuvant settings) or combination therapy with both anti-PD-1 and anti-CTLA4 (ipilimumab/nivolumab). Samples were taken at three different time points at routine blood draws and stored frozen in our biobank before processing. Blood samples were collected before initiation of ICI, hereafter referred to as “naive”, to determine baseline levels of immune cells in the peripheral blood as well as cytokine levels in the plasma. After initiation of ICI, patients were clinically supervised with regular checkups, and a follow-up blood draw was performed after six months under ICI treatment, referred to as “6mo ICI”. If patients presented with cutAEs within the monitoring time, patients received additional checkups with blood draws, and changes in the immune signature during the acute phase of a cutAE, named “cutAE”, were analyzed. Routine follow-up was carried out according to current guidelines, including radiological assessment via PET/CT scans every 3 months (or earlier in the case of clinical evidence for disease progression). After 6 months (or earlier in the case of progressive disease leading to therapy modification or disease-associated death), responses to therapy were categorized as responders or non-responders (using RECIST criteria). Patients with no evidence of disease in an adjuvant setting and complete and partial responses in a therapeutic setting were classified as responders. In contrast, patients with progressive disease were defined as non-responders.

### 2.2. Plasma Analysis and PBMC Isolation

Blood samples were collected in EDTA tubes and centrifuged without a break for plasma collection. After centrifugation, plasma was collected and stored at −20 °C for further analysis. Cytokine content in the plasma of patients was analyzed using a multiplex Th cytokine bead-based immunosorbent assay (Biolegend, San Diego, CA, USA; Cat. No 741027). The panel in this LegendPlex contains the following cytokines: IFN-γ, IL-5, TNF-α, IL-2, IL-6, IL-4, IL-10, IL-9, IL-17A, IL-17F, IL-22 and IL-13.

The remaining blood was diluted 1:4 with 1x PBS and layered over Ficoll. After centrifugation for 15 min at 1000 rpm without a break, the interface containing the mononuclear cells (PBMCs) was isolated and washed twice with RPMI 1640 (Biochrom, Berlin, Germany). PBMCs were counted and stored at −80 °C in cryo-preservation medium containing FCS (CH 30160.03 Hyclone Perbio, Fisher Scientific, Munich, Germany) and 10% DMSO (Carl Roth, Karlsruhe, Germany).

### 2.3. Flow Cytometry

To determine different cell populations by flow cytometry, multicolor staining of PBMCs was performed. For this, 2 × 10^5^ cells per stain were plated into a round-bottom plate and incubated with human TruStain FcX^TM^ for Fc receptor blocking. The following antibodies were used for the analysis of the immune cell composition: anti-CD3-FITC (clone 3.9), anti-CD4-PB (clone Okt 04), anti-CD8-PerCP/Cy5.5 (clone SK1), anti-αβTCR-APC (clone IP26), anti-γδTCR-PE/Cy7 (clone 11F2, BD Bioscience, Heidelberg, Germany), anti-CD86-PE (clone BU63), anti-CD69-BV510 (clone FN50), anti-CLA-PerCP/Cy5.5 (clone HECA-452), anti-CCR5-PE (clone 2D7, BD Bioscience, Heidelberg, Germany), anti-CXCR3-APC (clone G02H7), anti-CCR8-PE (clone L263G8), anti-CCR3-APC (clone 5e.8), anti-CCR4-APC (clone L291H4), anti-CCR6-PE (clone G034E3), and anti-CCR10-PE (clone 6588-5). If not stated otherwise, all antibodies were purchased from Biolegend, San Diego, USA. Flow cytometry data were acquired using a FACSCanto II (BD Biosciences, Heidelberg, Germany) or Cytoflex LX (Beckman Coulter, Munich, Germany) and analyzed with FlowJo software version 10.9.0 (Tree Star, BD Biosciences). A detailed gating scheme can be found in Supplementary Figure S1A.

### 2.4. Statistics

If not otherwise stated, data are presented as the mean ± SEM. Statistical analysis of the data was performed with unpaired Student’s *t*-test (2-tailed) or with 2-way repeated-measures ANOVA and Tukey’s multiple-comparisons test. Analysis was performed with GraphPad Prism software version 9.5.1. *p* values less than 0.05 were considered statistically significant and are marked as follows: *: *p* < 0.05; **: *p* < 0.01; ***: *p* < 0.005.

## 3. Results

### 3.1. Clinical Characteristics of the Patient Cohort

Between September and October 2019, 30 patients (12 females and 18 males) with stage III/IV malignant melanoma were enrolled in this study with a mean age of 66.6 (±13.3) years. Detailed clinical characteristics are summarized in Table 1.

**Figure 1:**
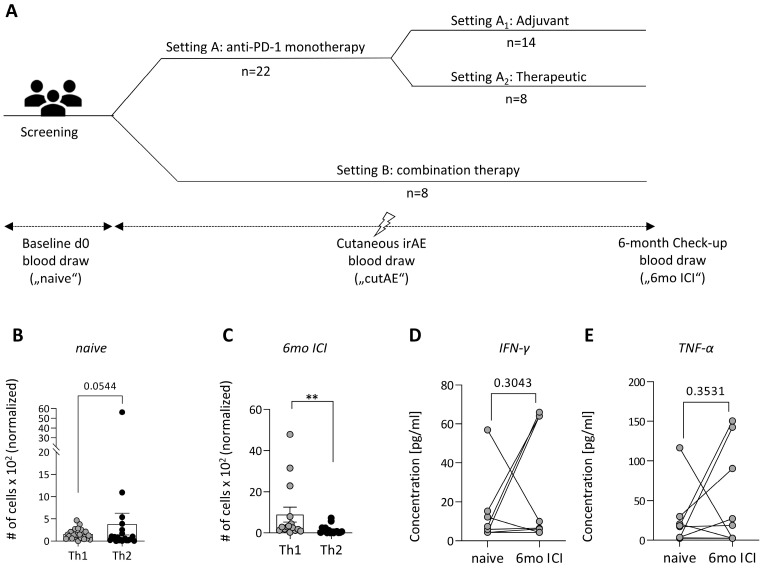
Study design and Th1/Th2 immune profile of patients. (**A**) Clinical study design. (**B**) Th1 and Th2 cells in melanoma patients’ PBMCs prior to ICI (naive) and (**C**) after 6 months of treatment (6mo ICI) were analyzed by flow cytometry (*n* = 22, mean with SEM, students *t*-test with Mann–Whitney correction, ** *p* < 0.01). (**D**) Plasma levels of Th1-associated cytokines IFN-γ and (**E**) TNF-α over the course of ICI (*n* = 15, Student’s paired *t*-test).

The majority (20 patients) were treatment naive. The other 10 patients received pre-treatment with targeted therapy (BRAF/MEK inhibition), interferon alpha, radiotherapy, or a combination, which was followed by disease progression. One patient also received chemotherapy with dacarbazine (three cycles) followed by targeted therapy. In 13 patients, BRAF V600E, in three patients, BRAF V600K and in one patient, BRAF G466E mutation were detected. PD-L1 was expressed by at least 1% of tumor cells in 14 patients. In 11 patients, the expression was lower than 1% and in 5 patients, the PD-L1 status was unknown. The schematic outline of the study setup is depicted in Figure 1A. Eight patients received ipilimumab/nivolumab combination therapy, fourteen patients were treated with PD-1 monotherapy in an adjuvant and eight patients received PD-1 in a therapeutic setting. The clinical response of the patients to ICI was determined and the cohort was stratified into responders and non-responders post hoc. During therapy, a complete response was achieved in four, a partial response in two and no evidence of disease in nine patients (summarized as responders). Fifteen patients showed progressive disease (five in adjuvant PD-1 monotherapy setting), defined as being non-responders.

Within the monitoring time, cutAEs occurred in twelve patients (40%). CutAEs were observed in 32% of patients receiving anti-PD-1 monotherapy, of which six received it in an adjuvant (43%) and one in a therapeutic setting (12.5%). However, 50% of patients receiving anti-CTLA-4/anti-PD-1 combination therapy presented with cutAEs. In total, 53% of patients responding to ICI (classified hereafter as responders, R) presented with cutAEs in our cohort, while only three non-responding patients (20%) showed signs of cutAEs. The observed severity of cutAEs was mild or moderate (grade 1 or 2 according to Common Terminology Criteria for Adverse Events, CTCAE v5.0) and patients were treated exclusively with topical steroids and oral antihistamines. We observed three cases of urticaria, acantholytic dermatosis and maculopapular exanthema each and two cases of newly occurred lichenoid dermatitis and psoriasis each. One patient developed urticaria and maculopapular exanthema at different time points.

To understand immune changes underlying cutAEs, PBMCs of patients were analyzed by flow cytometry and plasma levels of cytokines were determined (Supplementary Figure S1A). Melanoma patients before treatment showed a Th2 dominance in their immune profile, which shifted towards a Th1-dominated immune profile under ICI (Figure 1B,C). In line with these findings, an increase in plasma-level concentrations of Th1 hallmark cytokines IFN-γ and TNF-α was observed over the course of ICI treatment in many patients (Figure 1D,E).

### 3.2. Shift towards Th1 Cells in PBMCs during ICI Is Not Associated with a Favorable Clinical Response

**Figure 2:**
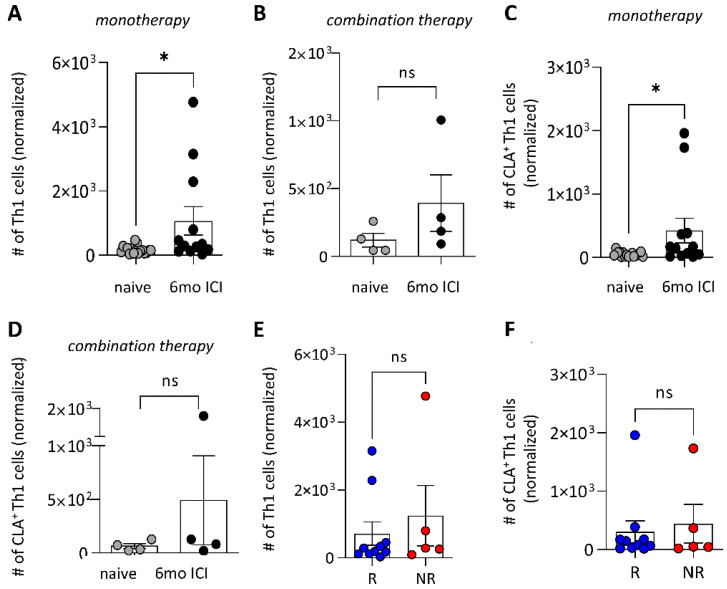
Shift towards Th1 cells in PBMCs during ICI. T-cell subsets in patients’ blood were analyzed by flow cytometry to define CD3^+^CD4^+^CXCR3^+^ Th1 cell numbers in patients receiving (**A**) anti-PD1 monotherapy (*n* = 19, mean with SEM, paired *t*-test with Welch’s correction, * *p* < 0.05, ns *p* > 0.05) or (**B**) anti-PD1/anti-CTLA4 combination therapy (*n* = 4, mean with SEM, paired *t*-test with Welch’s correction, ns *p* > 0.05). CD3^+^CD4^+^CXCR3^+^ CLA^+^ skin-homing Th1 cells were analyzed in patients receiving (**C**) anti-PD1 monotherapy (*n* = 19, mean with SEM, paired *t*-test with Welch’s correction, * *p* < 0.05) or (**D**) anti-PD1/anti-CTLA-4 combination therapy (*n* = 4, mean with SEM, paired *t*-test with Welch’s correction, ns *p* > 0.05). (**E**) Comparison of Th1 cells between responders (R, blue, *n* = 9) and non-responders (NR, red, *n* = 5) and (F) CD3^+^CD4^+^CXCR3^+^CLA+ skin-homing Th1 cells between responders (R, blue, *n* = 9) and non-responders (NR, red, *n* = 5) (mean with SEM, unpaired *t*-test with Welch’s correction, ns *p* > 0.05).

Anti-PD-1 monotherapy restored immune responses, which was reflected by an increase in Th1 cells in the peripheral blood of melanoma patients throughout six months of treatment (Figure 2A), which was not observed in the four patients analyzed receiving combination therapy with ipilimumab (Figure 2B). Patients under combination therapy had a 10-fold lower number of Th1 cells than those receiving monotherapy (Figure 2B). Interestingly, this difference was also significant for Th1 cells carrying the cutaneous lymphocyte-associated antigen (CLA) (Figure 2C,D). Even though the time period in which we followed our patients is too short for an accurate assessment of the overall survival, we observed an obvious difference in the progression-free survival between responders and non-responders (*p* = 0.06), this difference between responders and non-responders under immunotherapy was not due to the number of Th1 cells, nor CLA^+^ Th1 cells (Figures S1B and Figure 2E,F).

### 3.3. CutAEs Show a Th17/Th22-Related Immune Profile

**Figure 3:**
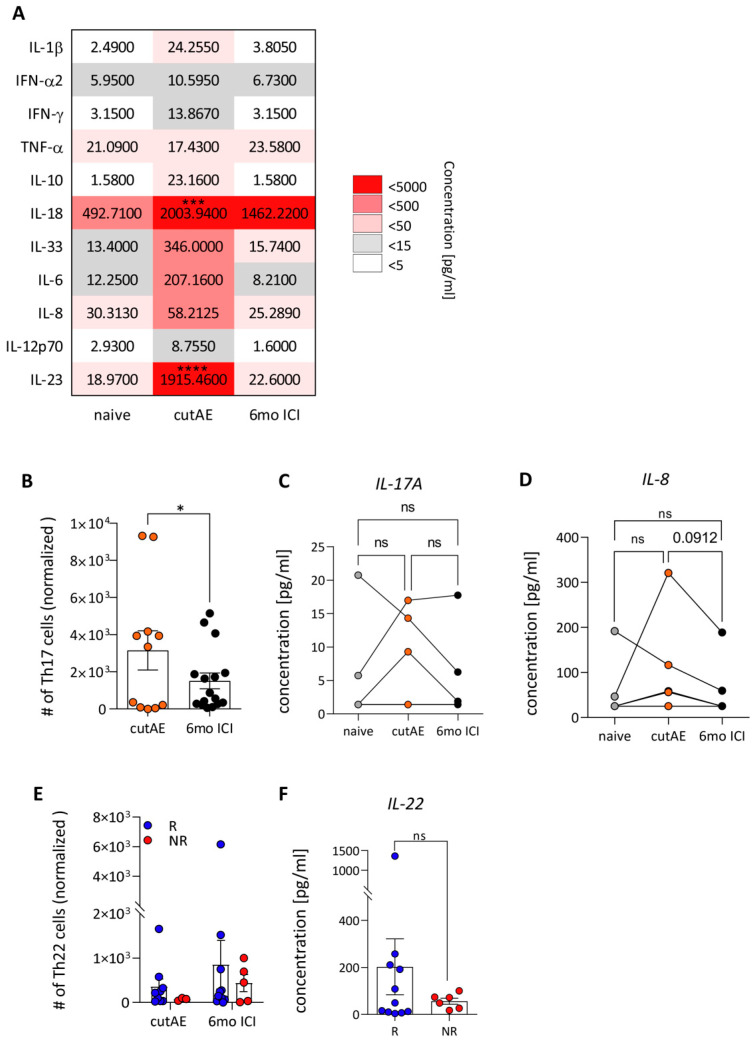
CutAEs show a Th17/Th22-related immune profile. (**A**) Cytokines in the plasma of patients were measured with an enzyme-linked immunosorbent assay before ICI (“naive”), during a cutAE (“cutAE”) and after 6 months of therapy (“6mo ICI”). Heatmap representation of median concentrations given in pg/mL. A color gradient is depicted from 0 to 5000 pg/mL (*n* = 30, 2way ANOVA, *** *p* < 0.005; **** *p* < 0.001. (**B**) Flow cytometry analysis of PBMCs for CD3^+^CD4^+^CCR4^+^CCR6^+^ Th17 cells (*n* = 10, mean with SEM, paired *t*-test, * *p* < 0.05). Plasma level analysis of (**C**) IL-17A and (**D**) IL-8 in patients (*n* = 7, paired *t*-test, ns *p* > 0.05). (**E**) Flow cytometry analysis of PBMCs for CD3^+^CD4^+^CCR10^+^ Th22 cells in responders (R, blue, *n* = 11) and non-responders (NR, red, *n* = 6) (mean with SEM, 2-way ANOVA). (**F**) Plasma levels of IL-22 during a cutAE between responders (R, blue, *n* = 11) and non-responders (NR, red, *n* = 6) (mean with SEM, unpaired *t*-test, ns *p* > 0.05).

Next, we explored changes in the plasma levels of inflammatory cytokines during an episode of a cutAE to determine potential biomarkers of cutAEs during ICI therapy. As expected, baseline levels of cytokines before ICI showed only low concentrations of inflammatory marker cytokines (Figure 3A). During the acute phase of a cutAE, however, an increase in plasma concentrations was measured. As expected, the type 2 alarmin IL-33 was elevated during a cutAE; however, interestingly, the cytokines IL-18 (*p* < 0.005) and IL-23 (*p* < 0.001) were significantly elevated in the plasma of patients during cutAEs. While all cytokine levels went back to baseline following the resolution of a cutAE, IL-18 levels remained high for the remaining study duration, showing a 4-fold higher concentration than in the naive state (Figure 3A). The cytokine IL-23 is a key mediator of the IL-23/IL-17 pathway and is pivotal in some inflammatory skin diseases. The 100-fold increase in IL-23 in the plasma indicates that the Th17/Th22 pathway is involved in cutAEs. In line with this, the patient cohort showed a significant increase in CD3^+^CD4^+^CCR4^+^CCR6^+^ Th17 cells during an acute cutAE, as well as a trend for an increase in the associated pro-inflammatory cytokines IL-17A and IL-8 (*p* = 0.09) during cutAEs in the plasma of patients (Figure 3B–D). This difference was not as substantial for CD3^+^CD4^+^CCR10^+^ Th22 cells, although responders showed a trend towards higher numbers of Th22 cells in the peripheral blood during an acute cutAE, maintaining this level during therapy (Figure 3E and Supplementary Table S2). This difference was also backed up by the tendency towards higher plasma levels of IL-22, which were four times higher in responders than non-responders (Figure 3F).

### 3.4. Increase in Inflammatory Cytokines at the Occurrence of cutAEs

**Figure 4:**
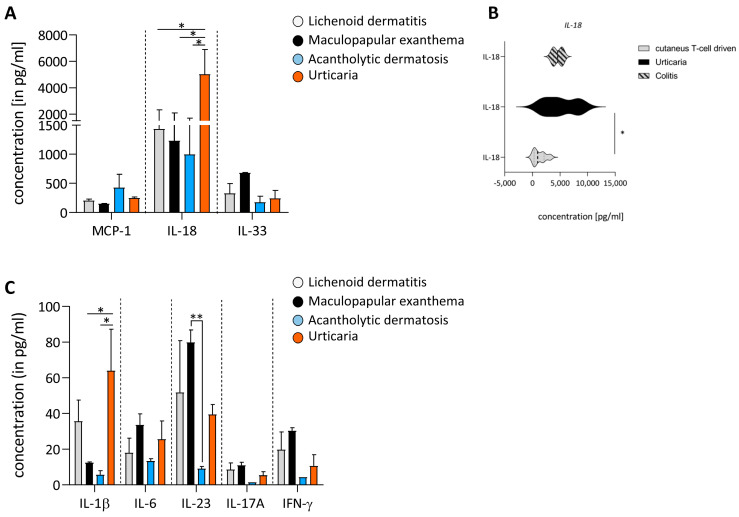
Cytokine profile of different cutAEs. (**A**) Plasma levels of inflammation-associated cytokines MCP−1 (CCL2), IL−18 and IL−33 in different subtypes of cutAEs (lichenoid dermatitis = grey, *n* = 2; maculopapular exanthema = dark grey, *n* = 3; acantholytic dermatosis = blue, *n* = 3; urticaria = orange, *n* = 3). Significantly higher plasma levels of IL-18 in urticaria (mean with SEM, 2-way ANOVA, ** *p* < 0.01). (**B**) Plasma levels of IL−18 in T-cell-driven cutAEs (light grey, *n* = 7) compared to mast-cell-driven urticaria (black, *n* = 3) and gastric colitis (stripped, *n* = 2) (mean with SEM, 2-way ANOVA, * *p* < 0.05). (**C**) Plasma levels of Th17-associated cytokines IL−1β, IL−6, IL−23, IL−17A and IFN−γ in different subtypes of cutAEs (lichenoid dermatitis = grey, *n* = 2; maculopapular exanthema = dark grey, *n* = 3; acantholytic dermatosis = blue, *n* = 3; urticaria = orange, *n* = 3) (mean with SEM, 2-way ANOVA, * *p* < 0.05).

One of the aims of this study was to investigate in more detail the underlying immunological differences between various cutAEs. Therefore, at the onset of cutAEs, patients were grouped based on the clinical manifestation, namely T-cell-mediated maculopapular exanthema, lichenoid dermatitis, acantholytic dermatosis and mast cell-driven urticaria. Immune signatures of plasma inflammatory cytokines were compared between the different clinical manifestations. Comparing the different inflammatory skin diseases showed significantly increased concentrations of IL-18, especially in patients diagnosed with urticaria (Figure 4A).

IL-18 is released by cells upon the activation of the inflammasome [15]. It is known that IL-18 activates MCs and can lead to the formation of mucosal mastocytosis [16]. Furthermore, MCs play a key role in inflammatory bowel disease including Crohn’s disease [17]. To determine whether MC-dependent adverse events have similar underlying immune mechanisms, we compared the IL-18 plasma levels of patients with the cutAE subtype urticaria to patients with T-cell-driven cutaneous AEs and colitis. Interestingly, it became apparent that the inflammatory cytokine profile of urticaria resembles that of colitis more closely than that of other cutAEs (Figure 4B). The inflammasome-related cytokine IL-1β was also significantly upregulated in patients with urticaria (Figure 4C). As shown before, patients show dominance in Th17 cells during cutAEs. The highest level of the pro-inflammatory Th17-associated cytokine IL-23 was detected in maculopapular exanthemas, while in acantholytic dermatosis, it was reduced compared to maculopapular exanthemas (Figure 4C).

### 3.5. Pro-Inflammatory IL-10 Is Associated with cutAEs and Increased in Non-Responders

**Figure 5:**
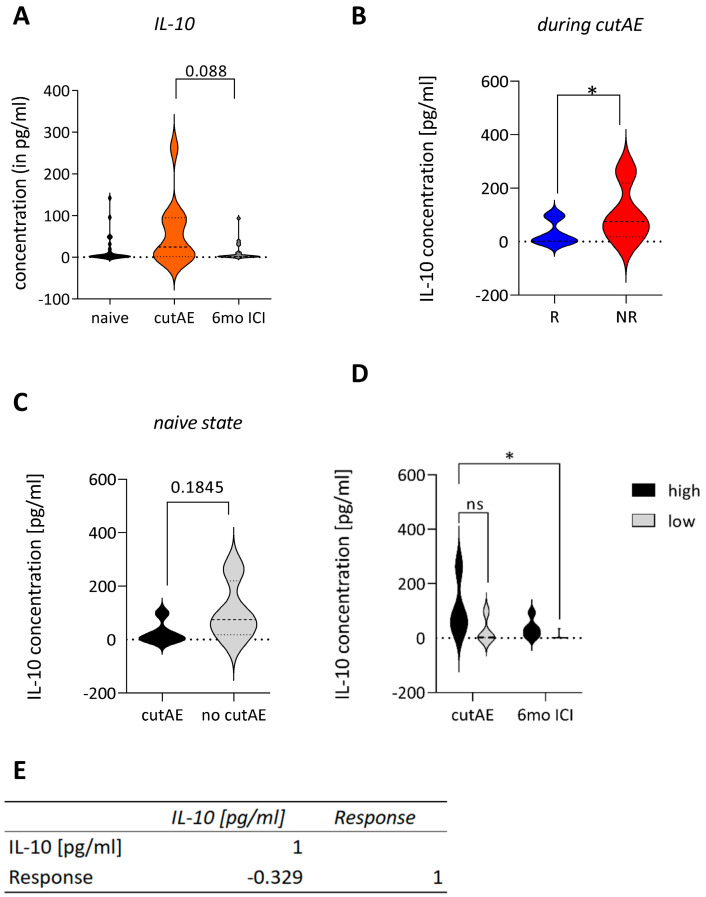
IL−10 as a prognostic marker. (**A**) High plasma IL−10 in patients during cutAEs (*n* = 24, mean with SEM, paired *t*-test with Wilcoxon correction). (**B**) Higher plasma IL−10 levels in non-responding patients during cutAEs (*n* = 11, mean with SEM, unpaired *t*-test * *p* < 0.05). (**C**) High naive plasma levels of IL−10 in patients who developed cutAEs as compared to patients not developing cutAEs during ICI. (**D**) Naive patients were grouped as IL−10^high^ and IL−10^low^ patients and the development of IL−10 in the plasma was analyzed during ICI. naïve IL−10^high^ patients show a tendency to have higher IL−10 concentrations during cutAEs (*n* = 11, mean with SEM, 2-way ANOVA, * *p* < 0.05, ns *p* > 0.05). (**E**) Pearson correlation analysis of baseline IL−10 levels (in pg/mL) and response. Pearson’s r-values as indicated in the table (*p* < 0.09).

IL-10 is a potent anti-inflammatory cytokine, which can be produced by many cell types including melanoma cells and Tregs, thereby playing an essential role in dampening the immune responses and inhibiting pro-inflammatory signals [18,19]. In our cohort, plasma levels of IL-10 were elevated during cutAEs, which may reflect a counter-regulation against skin inflammation associated with cutAEs (Figure 5A). The highest concentration of IL-10 was detected in patients 20, 25, 27, and 30. Each patient presented with a different cutAE, indicating no direct connection between IL-10 and a specific subset of cutAE. However, the difference in IL-10 was significantly higher when comparing all responders to all non-responders, potentially because the immune system is inhibited by IL-10 (Figure 5B). Patients might be predisposed to develop cutaneous inflammation, as baseline IL-10 levels tended to be lower in patients who later developed cutAEs (Figure 5C). Patients were therefore grouped into IL-10^high^ and IL-10^low^, based on their baseline IL-10 levels measured before initiation of ICI with a cut-off value of 1.58 pg/mL. Patients with high baseline IL-10 levels before ICI maintained these high IL-10 levels upon the appearance of cutAEs and at the 6-month follow-up, while the IL-10 levels of patients with low IL-10 levels at baseline nearly vanished throughout the course of ICI (Figure 5D). High levels of IL-10 in patients before ICI correlated negatively with the response to therapy (*p* < 0.09), supporting that a pre-existing pro-tumoral immune environment can influence the outcome of therapy (Figure 5E).

## 4. Discussion

ICI has become a pillar of melanoma therapy, but the immunological consequences of ICI have so far yet to be adequately investigated. It has been shown that cytotoxic irAEs vary based on the tumor entity, even when patients are treated with the same immunotherapeutic agent [20]. ICI primarily aims to enhance the cytotoxic response by CD8^+^ T cells or NK cells. Other studies have mainly concentrated on PD1^+^ T-cell subsets as response markers for clinical responses associated with rescuing exhausted CD8^+^ effector T cells by ICI. This pilot study aimed to identify immune cell subsets and cytokines responsible for different cutAEs in melanoma patients and to define potential biomarkers.

A recent study analyzing common immunological states preceding distinct manifestations of ICI-induced toxicity identified two baseline features essential for the determination of ICI-induced cutAEs: the abundance of activated CD4^+^ memory T cells and a clonally diverse TCR repertoire in the peripheral blood [21]. Therefore, the basis of this study was the analysis of CD4^+^ T helper subsets in the peripheral blood of melanoma patients, as well as T-cell-specific cytokine profiling. In our patient cohort, a shift from Th2 towards Th1 cells was induced through ICI in all patients irrespective of response to ICI therapy. Skin-homing CLA^+^ Th1 cells migrate to the skin via their homing receptor CLA and recirculate between the blood and skin. The significant increase in CLA^+^ Th1 cells in the peripheral blood of patients during monotherapy mirrors an increased recruitment of skin-associated T cells to the site of inflammation. Even though it burdens the patient, cutAEs are promising prognostic biomarkers for a competent immune system.

During ICI, an increase in inflammatory cytokines was observed in the plasma of patients, which were elevated even further during cutAEs. In particular, IL-1β, IFN-γ, IL-33, IL-6, IL-18 and IL-23 were strongly elevated during cutAEs, which are associated with autoimmune reactions. This finding is in line with recent studies conducted on melanoma patients. In a longitudinal study, an increase in eleven circulating pro-inflammatory cytokines in patients with severe immune-related toxicities was detected. The expression of these 11 cytokines was integrated into a single toxicity score, which showed predictive utility and correlation with grade 3 or greater irAEs in melanoma patients receiving anti-PD1 ICI [22]. The increase in the type 2 alarmin IL-33 and in IL-6 was expected, as both cytokines are secreted during tissue damage [23,24].

In particular, the cytokines IL-6, IL-18 and IL-23 play an irreplaceable role in antitumor immunity and ICI resistance [25]. The systemic increase in plasma IL-6, IL-8 and IL-33 supports the differentiation of Th17 cells and Th22 cells from naive CD4^+^ T cells [26,27]. Indeed, a significant increase in Th17 cells was detected in the peripheral blood of patients during cutAEs, as well as a trend towards elevated plasma levels of IL-17A and IL-8. It was reported that Th17 cells were increased after CTLA-4 blockade in patients with metastatic melanoma. However, the lack of differences between responder and non-responder patients indicated that Th17 cells did participate in the response to therapy. Significantly, this increase in Th17 cells was driven mainly in patients with cutAEs, suggesting a possible role for Th17 cells in ICI-induced toxicities [28]. Similar results were obtained in another study performed in melanoma patients treated with ipilimumab, in which pre-treatment IL-17 levels were associated with the development of severe intestinal inflammation [29]. Th17 cells play an essential role in the defense against extracellular pathogens. Further, we saw a trend towards an increase in Th22 cells in our patients with the onset of cutAEs, particularly in responders, which also remained higher after 6 months. This was further supported by a 4-fold increase in the levels of IL-22 in the plasma of patients responding to ICI. IL-22 is primarily secreted by lymphoid cells, mainly group 3 innate lymphoid cells, NKT cells and CD4^+^ T helper cells [30]. IL-22 promotes barrier integrity and repair by mediating communication between the skin barrier and the immune system, thereby playing a pivotal role in skin homeostasis and inflammation. It has become clear, however, that Th17 cells can also secrete IL-22 [31]. Together with the described elevated pro-inflammatory cytokines, the Th17/Th22-related profile suggests a potentially ICI-induced autoreactive origin of the occurring cutAEs [32,33]. Identification of distinct cytokine profiles for specific clinical manifestations remained difficult due to low case numbers and the heterogeneity of cutAEs in this pilot study.

The immunological mechanisms underlying cutAEs have yet to be investigated in heterogeneous patient cohorts with ill-defined cutAEs described as “rashes” or “exanthemas”. However, some cytokine signatures in the current study were tailored to specific clinical manifestations, e.g., patients with urticaria show a significant increase in plasma concentrations of IL-18 and IL-1β compared to other cutAEs (Figure 4A,B). The plasma levels of IL-18 in urticaria patients are similar to those with colitis, the second most common irAE under ICI (Figure 4C). IL-18 is an immunoregulatory cytokine produced mainly by keratinocytes and epithelial cells. It has been shown to play a role in the pathomechanism of chronic urticaria [34]. IL-18 stimulates mast cells and basophils to produce mediators such as histamine and may thus foster the development of urticaria [35]. Additionally, the inappropriate release of IL-1β and IL-18 has been demonstrated to elicit urticaria in autoinflammatory syndromes. This suggests a potential ICI-induced “autoimmune bias” underlying the occurring skin diseases, which underlines that ICI-related cutAEs do not necessarily share single immunological patterns and must be analyzed separately. Further studies with more participants will be necessary to specify cytokine profiles of different cutAE subgroups and compare them to classical skin diseases.

IL-10 blocks the Th1 response and promotes Th2 responses [36,37]. Specifically, it inhibits the synthesis of IFN-γ and IL-2 and blocks the production of other immune-enhancing cytokines such as IL-8, TNF-α and IL-12 [38,39,40,41]. Interestingly, patients with NSCLC with high plasma IL-10 levels were found to have enriched numbers of Th17 helper cells and mast cells [42]. In our study, we saw a tendency towards elevated levels of IL-10 during cutAEs (*p* < 0.09), which was pronounced in the group of non-responders (*p* < 0.05). IL-10 is a potent anti-inflammatory cytokine that plays a central role in limiting the host’s immune response to pathogens, which could also be the case in cutAEs. However, on the other hand, this could also dampen the antitumor response. Elevated IL-10 levels have been reported to be correlated with poor survival [43,44]. Thus, increased IL-10 expression associated with cutAEs may be tumor-promoting and not beneficial for the patient. This may imply that cutAEs in melanoma, which induce an IL-10 response, should be treated fast and effectively to reduce cutAEs early and prevent further production of IL-10, as prolonged treatment with corticosteroids has been shown to blunt antitumor efficacy [45].

Limitations of this study include the single-center setting and the low number of patients, which may have prevented the finding of more significant differences. Furthermore, only a limited number of inflammatory cytokines and T-cell immune cell subsets were analyzed at three time points, and a broader approach, e.g., including effector T cells, may have led to more findings. However, the number of studies in this setting is limited, and those with larger numbers of patients are lacking [46,47,48]. Preliminary results in limited populations may pave the way for more directed and more extensive studies. This prospective study, designed as an exploratory study to be followed up by larger studies in the future, compared patients with individual clinical manifestations of cutAEs and provided several significant and near-significant results that warrant follow-up. This study’s strengths were the analysis of T-cellular mechanisms and cytokines not only before and at the onset of cutAEs but also after a six-month follow-up in melanoma patients, and cutAEs in these patients were clinically thoroughly classified and correlated with immune signatures.

## 5. Conclusions

In conclusion, the present study of peripheral blood T-cell subtypes and cytokines revealed a shift from Th2 towards Th1 cells and skin-homing CLA^+^ Th1 cells in all patients with ICI therapy. In cutAEs, distinct immune signatures with increased pro-inflammatory cytokines were identified. We deliver evidence for the involvement of the Th17/Th22 pathway in cutAE pathogenesis. Further, this pilot study highlighted that different cutAEs can manifest with distinct cytokine profiles, such as IL-18 and IL-1β being elevated in patients presenting with urticaria. Particularly in non-responders, we found elevated levels of IL-10 during cutAEs, which might mirror an anti-inflammatory counter-regulation but simultaneously could dampen the antitumor response. These findings may support the early and intensive treatment of cutAEs. Future investigations with larger patient populations are desirable to substantiate these findings.

## Figures and Tables

**Table 1:** Clinical characteristics of melanoma patients included in this study.

Patient	Gender/Age ^a^	Melanoma Subtype/Site/Breslow in mm ^b^	BRAF/PD-L1 ^c^	Involved Sites ^d^	Pre-Treatment ^e^	Treatment Group ^f^	Response ^g^	cutAE ^h^	cutAE Subtype ^i^	cutAE Grade ^j^
1	83/F	snSSM/T/1.1	V600E/unk	CUT	none	PD1-a	R	+	lichenoid dermatitis	1
2	80/M	NM/HN/1.9	WT/7%	LN	none	PD1-a	R	+	acantholytic dermatosis	1
3	81/F	NM/UE/6.8	WT/2%	CUT, LN	none	PD1-t	R	−		
4	39/M	NM/AN/>8	V600E/<1%	LN	none	PD1-a	R	−		
5	57/M	uNM/T/2.1	V600K/1%	LN	none	PD1-a	R	−		
6	66/M	unk/T/1	WT/<1%	LN, PUL, ST	none	combi	NR	+	acantholytic dermatosis	1
7	65/M	uNM/HN/1.9	G466E/2%	CUT, LN	none	combi	R	+	psoriasis	1
8	38/M	unk/UE/7	V600E/unk	LN, ST, PUL, HEP, OSS, CUT	DTIC, TT	combi	NR	−		
9	81/M	unk/LE/>8	WT/0%	LN, OSS	none	PD1-t	NR	−		
10	77/F	snSSM/LE/1.6	V600K/15%	CUT, LN, ADR, INT	RT, TT	PD1-t	NR	−		
11	68/M	unk/T/>8	V600E/2%	LN, CUT, HEP, OSS	none	PD1-a	NR	−		
12	64/F	unk/T/0.7	V600E/6%	LN, PUL	TT	PD1-t	R	−		
13	62/F	ALM/UE/8	V600E/2%	LN	IFN	PD1-a	NR	−		
14	62/M	PM/T/>8	V600E/0%	ST, HEP, ADR, PAN, LN, OSS	TT	combi	NR	+	maculopapular exanthema	2
15	88/F	uALM/UE/>8	WT/<1%	LN, ADR	RT	PD1-t	R	−		
16	78/M	LMM/HN/0.7	V600K/2%	LN, SC	none	PD1-t	NR	−		
17	48/M	ALM/UE/2.1	V600E/20%	LN, CUT	none	PD1-t	NR	−		
18	59/F	ALM/UE/4.9	WT/unk	CUT	none	PD1-a	NR	−		
19	62/M	uNM/UE/2.5	WT/1%	CUT	none	PD1-a	R	+	maculopapular exanthema	1
20	81/M	unk/T/2	WT/unk	SC, LN, PUL	RT, IFN	PD1-t	R	+	urticaria	1
21	77/M	uNM/UE/>8	WT/0%	LN	none	combi	NR	+	acantholytic dermatosis	1
22	51/F	SSM/T/1.1	V600E/unk	LN	IFN	PD1-a	R	+	urticaria	1
23	64/M	SSM/T/2	WT/0%	LN	none	PD1-a	R	−		
24	51/F	SSM/HN/1.5	V600E/<1%	LN, SC, ADR, BIL, OSS, ST	IFN	combi	NR	−		
25	84/M	snSSM/T/>8	WT/2%	PUL	none	combi	NR	−		
26	59/F	NM/T/>8	V600E/<1%	LN	none	PD1-a	NR	+	lichenoid dermatitis	1
27	76/M	uNM/T/4.9	WT/0%	LN	none	PD1-a	R	−		
28	74/M	NM/T/1.3	V600E/30%	LN	none	PD1-a	NR	−		
29	55/F	NM/UE/1.2	V600E/0%	LN, CUT, PUL, CER	IFN	combi	R	+	urticaria, maculopapular exanthema	2, 2
30	68/F	uNM/T/3.2	WT/60%	LN	none	PD1-a	R	+	psoriasis	1

^a^ gender: female (F) or male (M); patients’ age at the time of study inclusion. ^b^ melanoma subtype: ulcerated (u), secondary nodular (sn), superficial spreading melanoma (SSM), nodular melanoma (NM), acral lentiginous melanoma (ALM), lentigo maligna melanoma (LMM), polypoid melanoma (PM), subtype unknown (unk); site of primary tumor: head and neck (HN), upper extremity (UE), torso (T), lower extremity (LE), anal (AN); Breslow thickness in mm. ^c^ BRAF mutation (V600E, V600K, G466E) or wild type (WT); PD-L1 positivity expressed through tumor proportion score. ^d^ sites involved in metastasis: adrenal (ADR), gallbladder/bile (BIL), cerebral (CER), cutaneous (CUT), hepatic (HEP), intestinal (INT), lymph node (LN), skeletal (OSS), pancreatic (PAN), pulmonary (PUL), soft tissue (ST), subcutaneous (SC). ^e^ pre-treatment: targeted therapy with BRAF/MEK inhibitors (TT), radiotherapy (RT), interferon-α (IFN), dacarbazine (DTIC). ^f^ treatment group: PD1 monotherapy in adjuvant setting (PD1-a), PD1 monotherapy in therapeutic setting (PD1-t), ipilimumab/nivolumab combination therapy (combi). ^g^ Response: responder (R: no evidence of disease in adjuvant setting; complete response and partial response in therapeutic setting), non-responder (NR: progressive disease). ^h^ cutaneous immune-related adverse events: yes (+); no (−). ^i^ subtype of cutaneous immune-related adverse event. ^j^ grade of cutaneous immune-related adverse events according to CTCAE v5.0 (G1-5).

## Data Availability

The data sets generated during the current study are available from the corresponding authors upon reasonable request.

## Supplementary Materials

The following supporting information can be downloaded at https://www.mdpi.com/article/10.3390/cancers16061226/s1, Figure S1: Flow cytometry gating scheme and PFS of patients; Table S1: Inclusion and exclusion criteria for patients; Table S2: Immune cell proportions.

## Abbreviations

CLA	cutaneous lymphocyte-associated antigen
CT	computer tomography
CTLA-4	cytotoxic T-lymphocyte-associated protein 4
cutAE	cutaneous immune-related adverse event
DMSO	dimethylsulfoxide
EDTA	ethylenediaminetetraacetic acid
FCS	fetal calf plasma
ICI	immune checkpoint inhibition
irAE	immune-related adverse event
NK	natural killer cell
NR	non-responder
NSCLC	non-small cell lung cancer
PBMC	peripheral blood mononuclear cell
PBS	phosphate-buffered saline
PD1	programmed cell death protein 1
PET	positron emission tomography
PFS	progression-free survival
R	responder
RECIST	response evaluation criteria in solid tumors
