# Supplementary material for: T-Cell Subtypes and Immune Signatures in Cutaneous Immune-Related Adverse Events in Melanoma Patients under Immune Checkpoint Inhibitor Therapy
Source: Cancers (Basel). 2024 Mar 20;16(6):1226. doi: 10.3390/cancers16061226 (PMC10969757; doi:10.3390/cancers16061226)
Supplement: Supplementary file 1 [file cancers-16-01226-s001.zip › Supplementary Table S1.pdf]

Supplementary Table S1: Inclusion and exclusion criteria for patients

Inclusion Criteria	Exclusion Criteria
Age >18 years	Prior treatment with anti-PD-1 or anti-CTLA-4 therapy
Capable of providing written and informed consent	Switching to Braf/MEKi during the study course
Advanced malignant melanoma (stage III or IV)	
Treatment with either anti-PD-1 monotherapy or anti-PD-1/anti-CTLA-4 combination therapy according to recommendation of interdisciplinary tumor board	
