# Supplementary material for: T-Cell Subtypes and Immune Signatures in Cutaneous Immune-Related Adverse Events in Melanoma Patients under Immune Checkpoint Inhibitor Therapy
Source: Cancers (Basel). 2024 Mar 20;16(6):1226. doi: 10.3390/cancers16061226 (PMC10969757; doi:10.3390/cancers16061226)
Supplement: Supplementary file 1 [file cancers-16-01226-s001.zip › Supplementary Table S2.pdf]

Supplementary Table S2: Immune cell proportions

	average number of cells	
	naive	cutAE
αβ T cells	26223,14	40970,24
γδ T cells	1067,41	1447,99
$CD4^+$ T cells	9221,03	20014,64
$CD8^+$ T cells	2839,16	9228,38
Th17 cells	2536,53	2601,46
Th22 cells	177,11	267,95
