# Supplementary figures and images for: T-Cell Subtypes and Immune Signatures in Cutaneous Immune-Related Adverse Events in Melanoma Patients under Immune Checkpoint Inhibitor Therapy
Source: Cancers (Basel). 2024 Mar 20;16(6):1226. doi: 10.3390/cancers16061226 (PMC10969757; doi:10.3390/cancers16061226)

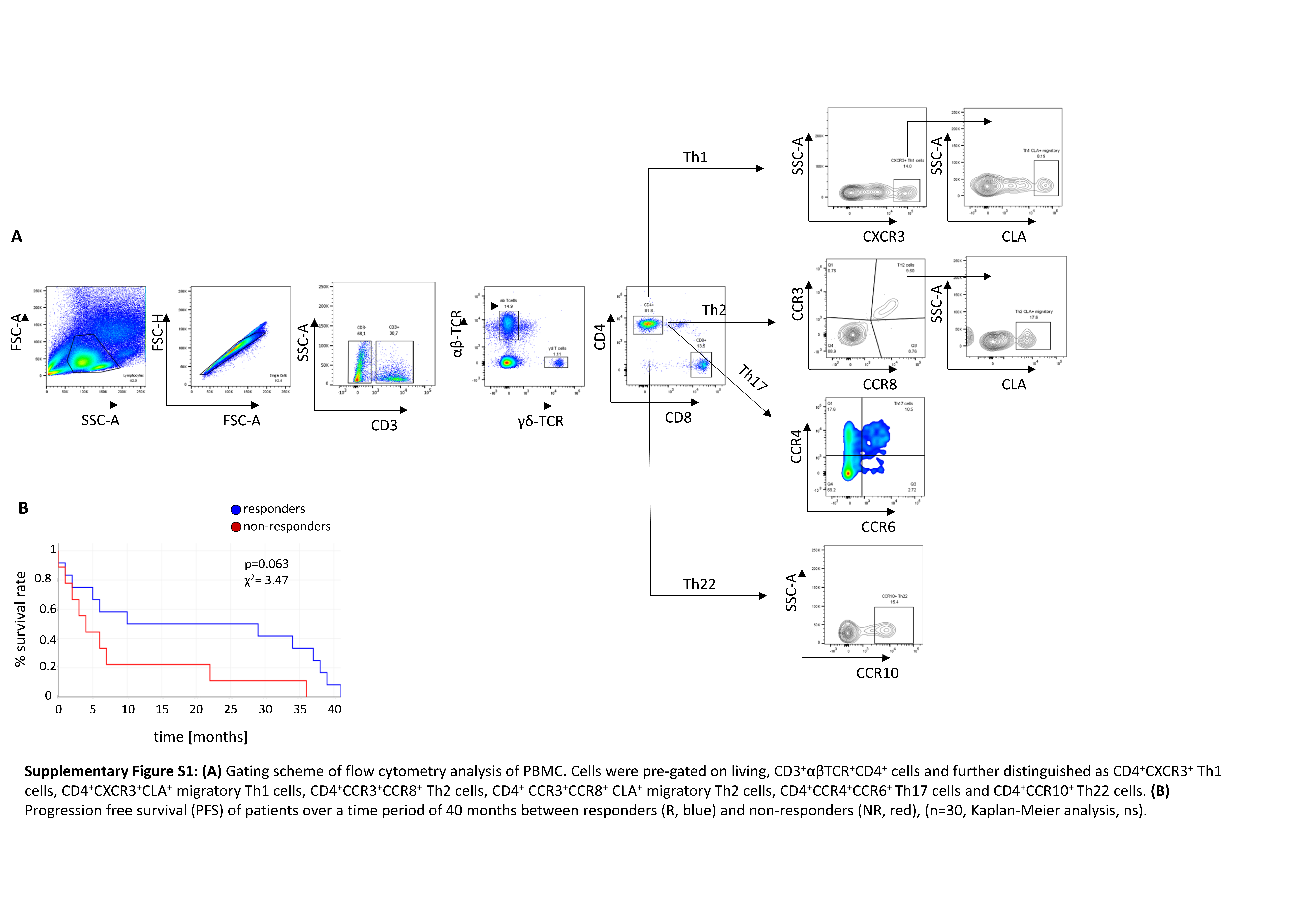

Supplement: Supplementary file 1 [file cancers-16-01226-s001.zip › Supplementary Figure S1.tif]
